# Clinical evaluation of Elecsys PIVKA-II for patients with advanced hepatocellular carcinoma

**DOI:** 10.1371/journal.pone.0265235

**Published:** 2022-03-10

**Authors:** Shun Kaneko, Masayuki Kurosaki, Kaoru Tsuchiya, Yutaka Yasui, Yuka Hayakawa, Kento Inada, Yuki Tanaka, Shun Ishido, Sakura Kirino, Koji Yamashita, Tsubasa Nobusawa, Hiroaki Matsumoto, Tatsuya Kakegawa, Mayu Higuchi, Kenta Takaura, Shohei Tanaka, Chiaki Maeyashiki, Nobuharu Tamaki, Yuka Takahashi, Hiroyuki Nakanishi, Namiki Izumi

**Affiliations:** Department of Gastroenterology and Hepatology, Musashino Red Cross Hospital, Tokyo, Japan; University of Campania "Luigi Vanvitelli", ITALY

## Abstract

**Background:**

Prothrombin induced by vitamin K absence-II (PIVKA-II) was reported as a diagnosis and prognosis marker for hepatocellular carcinoma (HCC). Although the development of systemic therapies for advanced HCC has been remarkable, the role of PIVKA-II is unclear. This prospective study aimed to verify Elecsys PIVKA-II compared with Lumipulse PIVKA-II in a cohort with advanced HCC undergoing systemic therapy.

**Methods:**

A total of 62 HCC patients who were treated with atezolizumab and bevacizumab (ATZ+BEV) and molecular targeted agents (MTAs) were prospectively enrolled at Musashino Red Cross Hospital from January 2020 to December 2020. A total of 208 serum samples from 52 patients were tested using Elecsys PIVKA-II and Lumipulse PIVKA-II assays. Furthermore, the relationship of Elecsys PIVKA-II and progression-free survival (PFS) was investigated with 48 patients (24 ATZ+BEV and 24 MTAs) whose Lumipulse PIVKA-II levels were >40 mAU/mL.

**Results:**

In the test accuracy analysis, the Elecsys assay has a correlation coefficient (R) of 0.92 compared with that of the Lumipulse assay (ATZ+BEV, 0.95; MTAs, 0.91). In the PFS analysis, the number of patients who received ATZ+BEV and MTAs as first- and late-line therapy were 9 and 13, and 15 and 11, respectively. The PIVKA-II response was defined for patients who had a reduction in the Elecsys PIVKA-II level on the first month of treatment evaluation. The PFS of patients with Elecsys PIVKA-II response was significantly longer than that of nonresponse patients (5.8 months vs 3.8 months, p = 0.0205).

**Conclusion:**

The Elecsys PIVKA-II was not only as useful as the Lumipulse PIVKA-II but also for stratifying the PFS of patients with advanced HCC.

## Introduction

Prothrombin induced by vitamin K absence-II (PIVKA-II) is an abnormal form of prothrombin secreted into the bloodstream when the activity of vitamin K-dependent carboxylase in the liver is inhibited as a result of the absence of vitamin K or the presence of vitamin K antagonists [1, 2]. In several studies, serum PIVKA-II was found to have a sensitivity, specificity, and accuracy of 48%–62%, 81%–98%, and 59%–84%, respectively, in diagnosing hepatocellular carcinoma (HCC) [3–5]. Some studies have shown that PIVKA-II was superior to other markers (alpha-fetoprotein [AFP] and lens culinaris agglutinin-reactive fraction of AFP [AFP-L3%]) in differentiating primary liver cancer from cirrhosis [6]. Additionally, it could determine whether suspicious liver nodules on ultrasonography were neoplastic or nonneoplastic [7]. According to recent data, the combination of the PIVKA-Ⅱ and AFP could significantly improve the diagnosis of HCC [8]. In patients with nonalcoholic steatohepatitis, the GALAD score, which determines the risk of HCC based on a patient’s sex, age, and serum levels of AFP, AFP-L3, and PIVKA-II, identified patients with any-stage HCC with an area under the curve (AUC) of 0.96 [9].

PIVKA-II was also reported as a prognosis marker in patients with HCC, as PIVKA-II and AFP have a positive correlation with the tumor burden [10]. High serum levels of PIVKA-II are associated with a more aggressive tumor behavior [11], the histologic grade of tumor differentiation [12], and macrovascular invasion (MVI) / portal vein tumor thrombosis (PVTT) [12–14]. PIVKA-II and AFP can also predict the recurrence of HCC and prognosis of HCC patients [15]. The prognostic implications of the expression patterns of three tumor markers, AFP, AFP-L3, and PIVKA-II, have been evaluated in patients with HCC. The overall survival (OS) and disease-free survival at 5 years postoperatively were significantly stratified by the number of positive markers [16]. Furthermore, the change in the PIVKA-II level during treatments can predict the prognosis of HCC patients [11]. It was also noted that the change in the PIVKA-II and AFP levels were positively associated with the radiological response in patients treated by transarterial chemoembolization (TACE) [17–19] and hepatic arterial infusion chemotherapy (HAIC) [20].

The development of drug therapies for advanced HCC that has remarkably progressed sorafenib [21] was approved for the treatment of unresectable HCC. The first-line therapy in the systemic treatment of advanced HCC dramatically has shifted from oral tyrosine kinase inhibitors to the combination therapy of atezolizumab and bevacizumab (ATZ+BEV). The ATZ+BEV was approved because of its ability to significantly prolong the progression-free survival (PFS) and OS in patients with unresectable HCC compared with that of sorafenib when used as first-line therapy [22]. Additionally, molecular targeted agents (MTAs) such as lenvatinib [23], regorafenib [24], cabozantinib [25], and ramucirumab [26] have been also approved for systemic therapy in patients with unresectable HCC. Although there have been reports on the effects of locoregional therapy such as TACE and HAIC, reports on the course of treatment and the behavior of PIVKA-II for ATZ+BEV and MTAs have been few.

The Elecsys PIVKA-II which was measured by a one-step sandwich assay with ECLIA and has been standardized against purified recombinant des γ carboxy prothrombin from cell culture, shows shorter assay time (18 minutes), simplicity, and potentially higher precision as compared with Lumipulse PIVKA-II, according to manufacture protocol. Moreover, the on-board stability of the Elecsys PIVKA-II reagent is 112 days, 82 days longer than that of Lumipulse PIVKA-II, and can be used without loss of reagent. Based on these points, Elecsys PIVKA-II may become useful widely if clinical practice evaluation is confirmed.

This prospective study aims to comparatively verify Elecsys PIVKA-II and conventional Lumipulse PIVKA-II from the serum samples of patients with unresectable HCC undergoing systemic therapy. We also investigated the ability of Elecsys PIVKA-II to reflect the clinical course of patients with unresectable HCC including comparison between ATZ+BEV and MTAs.

## Methods

The study protocol was approved by the Institutional Review Board and the Ethical Committee of Musashino Red Cross Hospital (No. 2062) and conducted following the Declaration of Helsinki (revised by Fortaleza in 2013) and Ethical Guidelines for Medical and Health Research Involving Human Subjects (partially revised on February 28, 2017). The written informed consent was obtained from all patients.

### 1. Patients and sample collections

A total of 62 HCC subjects who were scheduled for treatment with MTAs or immunotherapy were prospectively enrolled at Musashino Red Cross Hospital from January 2020 to December 2020. A series of four blood samples were collected from each patient during regular examination, and the first of the blood samples were drawn prior to any systemic therapy. The serum samples were divided into two tubes and stored at −20°C prior to measurement. Patient data, including age, sex, past history of hepatitis, blood test results, radiological imaging, survival, and PFS, were retrieved from the hospital medical records.

Inclusion criteria for chemotherapy were as follows: metastatic or locally advanced HCC that was unresectable or refractory to TACE with BCLC stage B or C and Eastern Cooperative Oncology Group performance status 0 to 1. Child-Pugh (C-P) classification, albumin–bilirubin (ALBI) [27], and mALBI grades [28] were used to assess liver function. The ALBI score was calculated on the basis of the serum albumin and total bilirubin values as previously reported. The ALBI grade was defined as the following: grade 1, ≤−2.60; grade 2, >−2.60 to ≤−1.39; and grade 3, >−1.39. Furthermore, ALBI grade 2 was divided into two subgrades (2a and 2b) using a cutoff value (−2.270), and such ALBI grades were defined as mALBI grades. The evaluation of radiologic response was evaluated according to the RECIST 1.1, as previously reported [29–31].

### 2. Assay of serum levels of PIVKA-II

The serum PIVKA-II level was determined using commercially available kits Lumipulse PIVKA-II-N (Lumipulse assay, Fujirebio, Tokyo, Japan) and Elecsys PIVKA-II (Elecsys assay, Roche Diagnostics K.K., Tokyo, Japan). Both tests were conducted following the instruction manuals of each kit.

### 3. Statistical analysis

Correlation was evaluated by the correlation coefficient (R) and linear regression. Chi-square and Fisher’s exact tests were used to compare categorical data. Student’s t-test or Mann–Whitney U test were used to analyze the distribution of continuous variables. Cumulative PFS rates of patients were prepared using the Kaplan–Meier method. The log-rank Mantel–Cox test was used to compare the cumulative PFS curves. *P* < 0.05 was considered statistically significant. JMP (v15.2.0 software, SAS Institute Inc., Cary, NC, USA), GraphPad Prism software (GraphPad Software, San Diego, CA, USA), and EZR (Saitama Medical Center, Jichi Medical University, Shimotsuke, Japan) were used to analyze statistical significance.

## Results

### 1. Trends of PIVKA-II levels in monitoring

**Fig 1:**
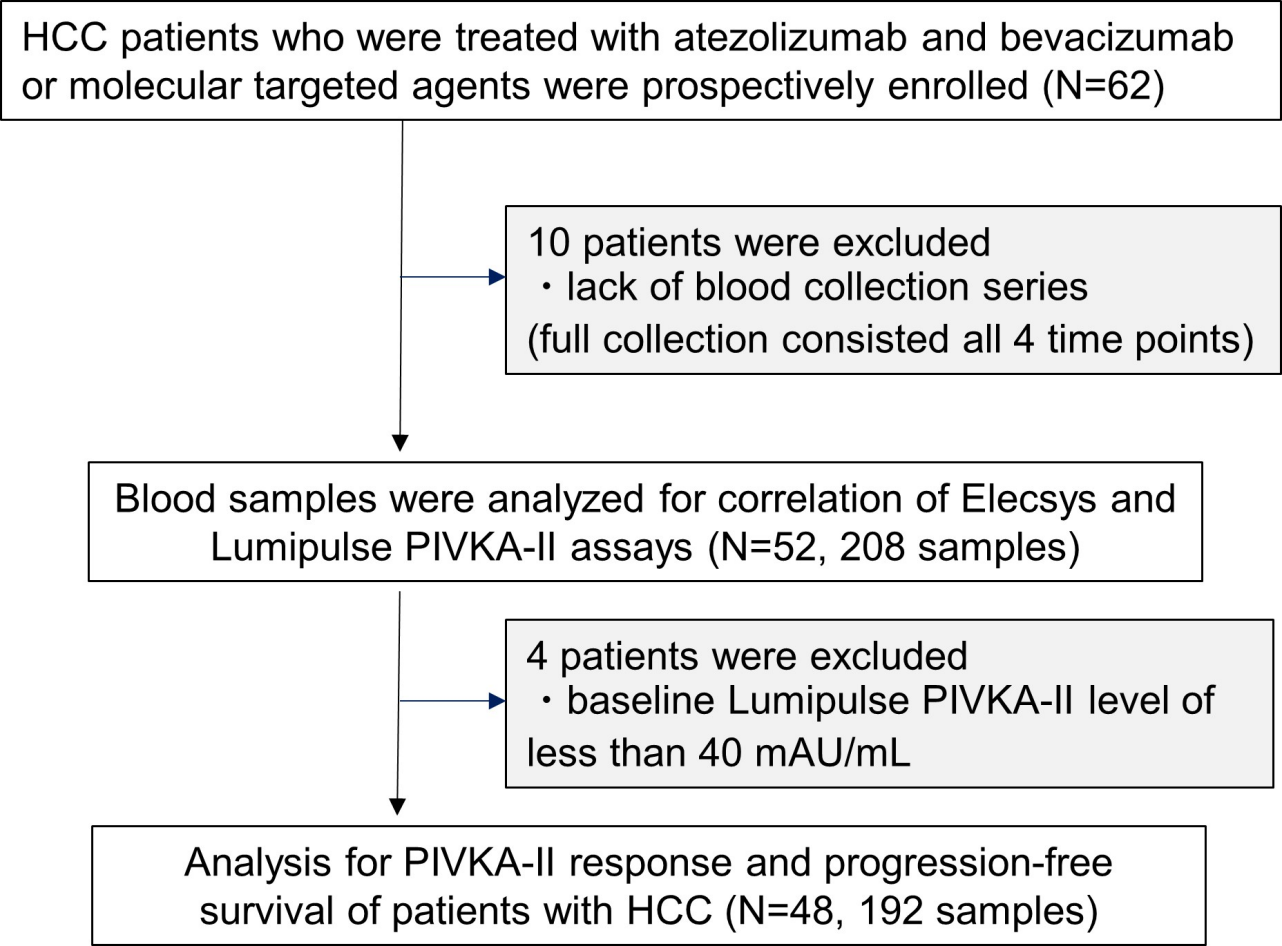
The flowchart of analysis.

**Fig 2:**
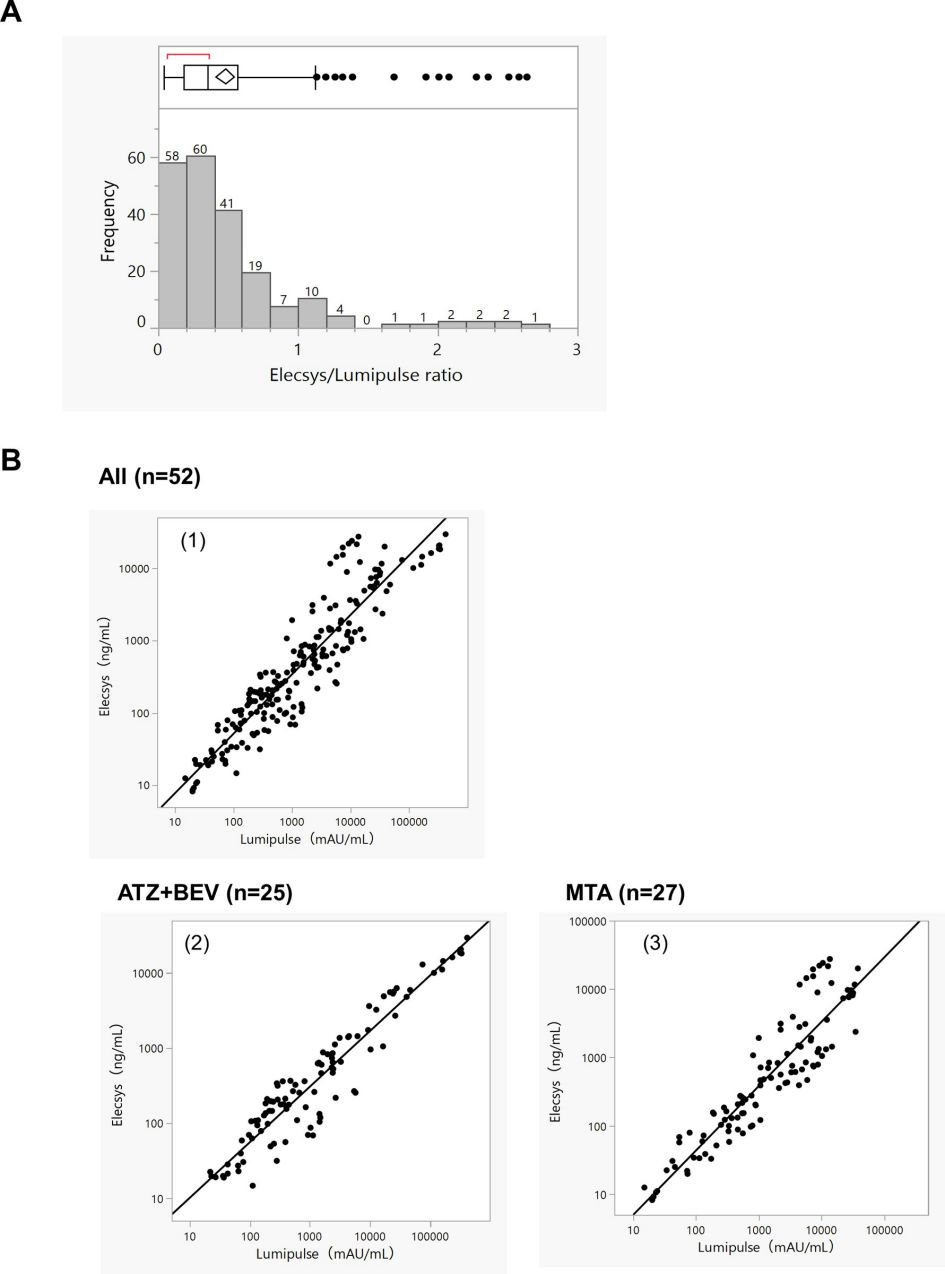
Analysis for the correlation of Elecsys and Lumipulse PIVKA-II assays. (A) The X-axis shows the Elecsys/Lumipulse ratio, and the Y-axis shows the number of samples. (B) Comparison of Elecsys and Lumipulse assays for the measurement of serum PIVKA-Ⅱ concentration. (1) All patients, (2) patients treated with atezolizumab and bevacizumab (ATZ+BEV), and (3) patients treated with molecular targeted agents (MTAs).

A total of 62 cases were enrolled in this study; 10 cases were excluded because of the lack of blood collection series. A total of 208 serum samples from 52 patients were tested using each assay (Fig 1). The trend of increase/decrease of the PIVKA-II level was compared between the Elescys and Lumipulse assays. The second to fourth blood collection from the 52 cases were categorized as “decrease” or “increase” if the sample was less or more than 100% of the previous value, respectively. The agreement rate in the trend of increase/decrease was 93.6% between the two assays. Ten samples had a discrepancy in the trend; however, the PIVKA-II level of these samples had minimal changes against that of the previous value, and the ratio to the previous value ranged from 88% to 115% and 89% to 154% for the Elescys assay and Lumipulse assays, respectively. The units of measurement for the Lumipulse and Elecsys assays were mAU/mL and ng/mL, respectively. Therefore, to study the conversion factor, we calculated the ratio between the measurements of the Elecsys and Lumipulse assays in each sample. The median was 0.36, and the interquartile range was 0.18–0.56 (Fig 2A).

### 2. Correlation of Elecsys and Lumipulse PIVKA-II assays

The correlation between the two assays was examined. The slope of the overall Elecsys assay and Lumipulse assay was 0.82 with an R of 0.92. Of the 52 patients, 27 patients received MTAs and 25 patients received ATZ+BEV. For the MTAs-treated and ATZ+BEV-treated groups, the slope and R of the Elecsys assay and Lumipulse assay were 0.94, R = 0.91 and 0.74, R = 0.95, respectively (Fig 2B). This confirms that the Elecsys assay is as useful as the Lumipulse assay.

### 3. Elecsys PIVKA-II response and PFS of patients with HCC

Furthermore, we investigated the relationship between Elecsys PIVKA-II and PFS. Patients who were excluded were those who had baseline Lumipulse PIVKA-II level of less than 40 mAU/mL (n = 4). Finally, 48 patients (24 ATZ+BEV and 24 MTAs) were included in this analysis (Fig 1). The baseline characteristics of the patients were shown in Table 1. Among them, 38 (79.2%) patients were male; the median age was 75 years (range, 23–86 years), and 47 (97.9%) patients were Child–Pugh A. The median ALBI score was −2.246. There were 27 (56.2%) and 21 (43.8%) patients with BCLC stages B (intermediate) and C (advanced), respectively. A total of 17 (35.4%) patients had extrahepatic metastasis (EM), and four (8.3%) had a major vascular invasion. The breakdown of patients regarding ATZ+BEV and MTAs therapy was nine and 13 cases as first-line and 15 and 11 cases as late-line, respectively. There was no significant difference between the patients who received ATZ+BEV and MTAs therapy.

**Table 1:** Clinical characteristics of patients with advanced HCC at baseline.

	All	ATZ + BEV	MTAs	p value
Number of patients	48	24	24	
Age (years), median (range)	75 (23–86)	74 (34–86)	75.5(23–86)	0.433
Sex: Male/Female (%)	38 (79.2%)/10 (20.8%)	20 (83.3%)/4 (16.7%)	18 (75.0%)/6 (25.0%)	0.724
Body weight (kg) <60/>60	23 (47.9%)/25 (52.1%)	13 (54.2%)/11 (45.8%)	10 (41.7%)/14 (58.3%)	0.564
Etiology of chronic liver disease				0.186
HBV	10 (20.8%)	5 (20.8%)	5 (20.9%)	
HCV	18 (37.5%)	10 (41.6%)	8 (33.3%)	
Alcohol	10 (20.8%)	7 (29.3%)	3 (12.5%)	
Others	10 (20.8%)	2 (8.3%)	8 (33.3%)	
Child–Pugh Class				0.999
A	47 (97.9%)	24 (100.0%)	23 (95.8%)	
B	1 (2.1%)	0 (0.0%)	1 (4.2%)	
ALBI score	−2.246 (−3.280 to −1.498)	−2.205 (−3.280 to −1.650)	−2.271 (−3.113 to −1.498)	0.951
mALBI grade 1/2a/2b/3	11/12/25/0	7/4/13/0	4/8/12/0	0.358
BCLC				0.999
B (intermediate stage)	27 (56.2%)	14 (58.3%)	13 (54.2%)	
C (advanced stage)	21 (43.8%)	10 (41.7%)	11 (45.8%)	
Extrahepatic spread				0.999
Yes	17 (35.4%)	8 (33.3%)	9 (37.5%)	
No	31 (64.6%)	16 (66.7%)	15 (62.5%)	
Macroscopic vascular invasion				0.999
Yes	4 (8.3%)	2 (8.3%)	2 (8.3%)	
No	44 (91.7%)	22 (91.7%)	22 (91.7%)	
AFP (ng/mL)	347.1 (0.9–38,288.6)	232.5 (4.8–19,776.9)	432.5 (0.9–38,288.6)	0.845
Lumipluse PIVKA-II (mAU/mL)	1280 (42–236,000)	753.5 (43–236,000)	2050 (42–29,700)	0.533
Elecsys PIVKA-II (ng/mL)	416.4 (14.65–16,220)	236.55 (14.65–16,220)	612.25 (30.56–9,536)	0.282
Chemotherapy regimen			SOR 6/LEN 13/REG 1/RAM4	
Treatment line 1st/2nd/3rd/and more		9/8/3/4	13/4/6/1	0.205

HCC, hepatocellular carcinoma; ATZ + BEV, atezolizumab and bevacizumab; MTAs, molecular targeted agents; HBV, hepatitis B virus; HCV, hepatitis C virus; ALBI, albumin–bilirubin; mALBI, modified ALBI; BCLC, Barcelona Clinic Liver Cancer; AFP, alpha fetoprotein; PIVKA-II, prothrombin induced by vitamin K absence-II; SOR, sorafenib; LEN, lenvatinib; REG, regorafenib; RAM, ramucirumab.

**Fig 3:**
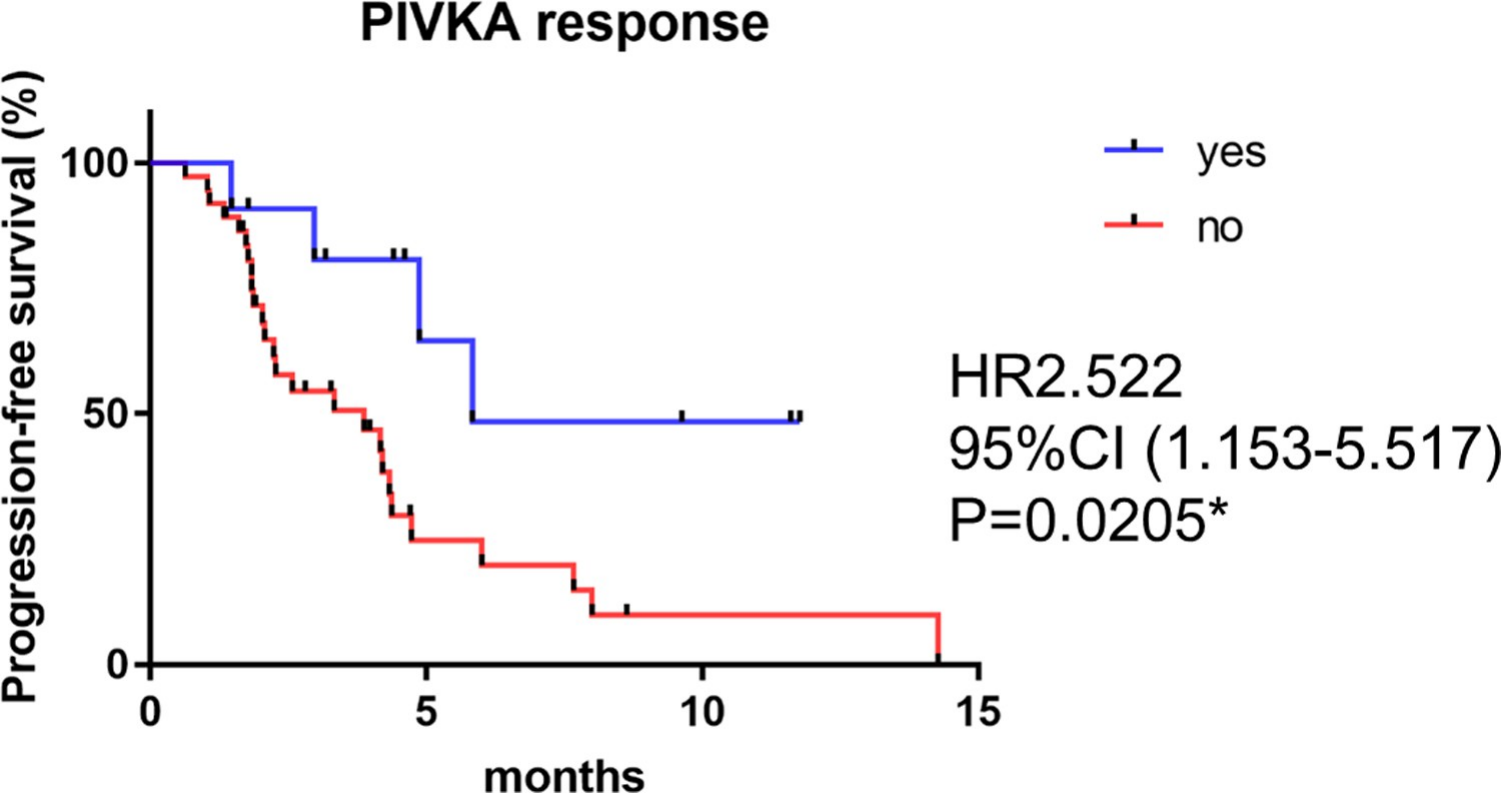
Cumulative progression-free survival (PFS) rates of advanced HCC patients treated by systemic therapy depending on Elecsys PIVKA-II response at first month.

The median PFS of patients treated with ATZ+BEV (n = 24) and MTAs (n = 24) were 4.2 and 4.2 months, respectively (log-rank test, *P* = 0.667). The PIVKA-II response was defined as patients who had a reduction in the Elecsys PIVKA-II level on the first month of treatment evaluation. The PFS of patients with Elecsys PIVKA-II response was significantly longer than that of nonresponse patients (5.8 vs 3.8 months, p = 0.0205) (Fig 3). The baseline factors were not significantly different between PIVKA-Ⅱ responders and non-responders as shown in Table 2. Furthermore, the PFS of response patients tended to be longer than that of nonresponse patients when the patients were divided into the ATZ+BEV and MTAs groups (S1 Fig).

**Table 2:** Clinical characteristics of PIVKA-Ⅱ responders and non-responders.

	Responder	Non- responder	p value
Number of patients	11	37	
Age (years), median (range)	71 (47–86)	76 (23–86)	0.376
Sex: Male / Female (%)	9 (81.8%)/2 (18.2%)	29 (78.4%)/8 (21.6%)	0.999
Child-Pugh Class			0.999
A	11 (100.0%)	36 (97.3%)	
B	0 (0.0%)	1 (2.7%)	
ALBI score	-2.562 (-3.280 to -2.008)	-2.230 (-3.113 to -1.498)	0.211
mALBI grade 1/2a/2b/3	3/3/5/0	8/9/20/0	0.905
BCLC			0.304
B (intermediate stage)	8 (72.7%)	19 (51.4%)	
C (advanced stage)	3 (27.3%)	18 (48.6%)	
Extrahepatic spread			0.723
Yes	3 (27.3%)	14 (37.8%)	
No	8 (72.7%)	23 (62.2%)	
Macroscopic vascular invasion			0.999
Yes	1 (0.9%)	3 (8.1%)	
No	10 (99.1%)	34 (91.9%)	
AFP (ng/ml)	753.7 (4.8–4323.6)	132.9 (0.9–38288.6)	0.508
Lumipluse PIVKA (mAU/mL)	1360 (42–236000)	1200 (43–29700)	0.646
Elecsys PIVKA (ng/mL)	366.7 (28.26–9536)	466.1 (14.65–16220)	0.885

ALBI, albumin–bilirubin; mALBI, modified ALBI; BCLC, Barcelona Clinic Liver Cancer; AFP, alpha fetoprotein; PIVKA-II, prothrombin induced by vitamin K absence-II.

## Discussion

This study provided valuable evidence that the Elecsys PIVKA-II was not only as useful as the Lumipulse PIVKA-II but also for stratifying the PFS of HCC patients treated with ATZ+BEV and MTAs. First, we investigated the relationship between Elecsys PIVKA-II and Lumipulse PIVKA-II as shown in Fig 1. The units of measurement for the Lumipulse and Elecsys assays are mAU/mL and ng/mL, respectively. Regarding the correlation between the two assays, the slope of the overall Elecsys assay and Lumipulse assay was 0.82 with an R of 0.92 (Fig 2). A good correlation between Elecsys and Lumipulse assays was seen with each ratio, which shows that the Elecsys PIVKA-II was as useful as the Lumipulse assay. On the other hands, PIVKA-II is an abnormal prothrombin molecule that lacks carboxylation (Gla) of some or all of the 10 glutamine residues (Glu) at the N-terminus of the prothrombin precursor, and is a complex substance with various forms depending on the degree of carboxylation [32]. It is suggested that it exists as a multiple PIVKA-II protein in various states with different numbers and sites of Glu even in the same patient. Therefore, there is a possibility that the population of PIVKA-II captured by using different assay systems may cause differences and it is difficult for the assay ratios of Elecsys and Lumipulse to converge to a consistent value.

Next, we investigated the utility of the Elecsys PIVKA-II level as a prognosis biomarker in patients with HCC during systemic therapy. PIVKA-II has been considered a useful marker for the diagnosis of HCC as well as AFP. However, it was controversial as a marker during systemic therapy. There are a few reports that have shown that the PIVKA-II response was related to OS and PFS in patients undergoing anti-PD-1 immunotherapy [33]. Other reports showed that a correlation with image evaluation was found in AFP but not in PIVKA-II in lenvatinib therapy [34]. This point largely depends on the timing of evaluation and the therapeutic agent. Conversely, an in vitro study showed that hypoxic stimulation-induced HCC cells (HepG2) produce PIVKA-II and become hypoxic [35]. Since hypoxia during the treatment process may affect the PIVKA-II level, it may be difficult to reflect only the progression of HCC, depending on the evaluation period. However, this point has not been confirmed in vivo and in clinical practice.

Regarding ATZ+BEV, its treatment results as a first-line drug have been shown in clinical trials [22], and it is currently used even after the second-line treatment in actual clinical practice. Recently, the efficacy and safety of ATZ+BEV have been shown in cases where it is used as a late-line treatment or those outside of the criteria of IMbrave150 [36, 37]. In this study, ATZ+BEV was administered to nine patients as first-line treatment and 15 patients as late-line treatment (Table 1). Although there are controversial opinions about the radiological response evaluation of HCC [38], we used RECIST, which is the most objective and reproducible evaluation for radiologic response in HCC [29–31].

Elecsys PIVKA-II was investigated for ATZ+BEV and MTAs; PFS was significantly prolonged in the response group (Fig 3). Although it was difficult to make a solid comparison because of the insufficient number of cases and observation period, we could compare the background characteristics between ATZ+BEV and MTAs, PIVKA-II responder and non-responder, respectively. They were not significantly different respectively, because of a well-designed prospective study (Tables 1, 2). This also indicates that PIVKA-II response is an important factor, though it is difficult to use only baseline factors to predict PFS.

The present study had several limitations. First, although the study showed the usefulness of Elecsys PIVKA-II in a cohort that included patients with advanced HCC, the study population was Japanese; hence, the results may not be generalizable because of racial differences. Second, the observation period and number of patients may be insufficient to arrive at definite comparisons and to evaluate the long-term survival. This study was only about PFS, and it would be desirable to consider whether it will lead to OS. Although the usefulness of PIVKA-II was demonstrated, it should be considered a complementary evaluation in combination with AFP. Combination therapy, such as ATZ+BEV, shows various behaviors; hence, sufficient evaluation is desired not only for image evaluation but also for tumor marker evaluation.

In conclusion, the Elecsys PIVKA-II was not only as useful as the Lumipulse PIVKA-II but also for stratifying the PFS for patients with advanced HCC. Understanding the responses of tumor markers may contribute to the appropriate evaluation and treatment of patients.

## Supporting information

S1 Fig Cumulative progression-free survival (PFS) rates of advanced HCC patients treated by atezolizumab and bevacizumab (ATZ+BEV) or molecular targeted agents (MTAs) depending on Elecsys PIVKA-II response at each time point. (A) PFS of patients treated by ATZ+BEV depending on Elecsys PIVKA-II response at 3, 6, and 9 weeks. (B) PFS of patients treated by MTAs depending on Elecsys PIVKA-II response at 2, 4, and 8 weeks.
(TIF)
