# Supplementary figures and images for: Clinical evaluation of Elecsys PIVKA-II for patients with advanced hepatocellular carcinoma
Source: PLoS One. 2022 Mar 10;17(3):e0265235. doi: 10.1371/journal.pone.0265235 (PMC8912231; doi:10.1371/journal.pone.0265235)

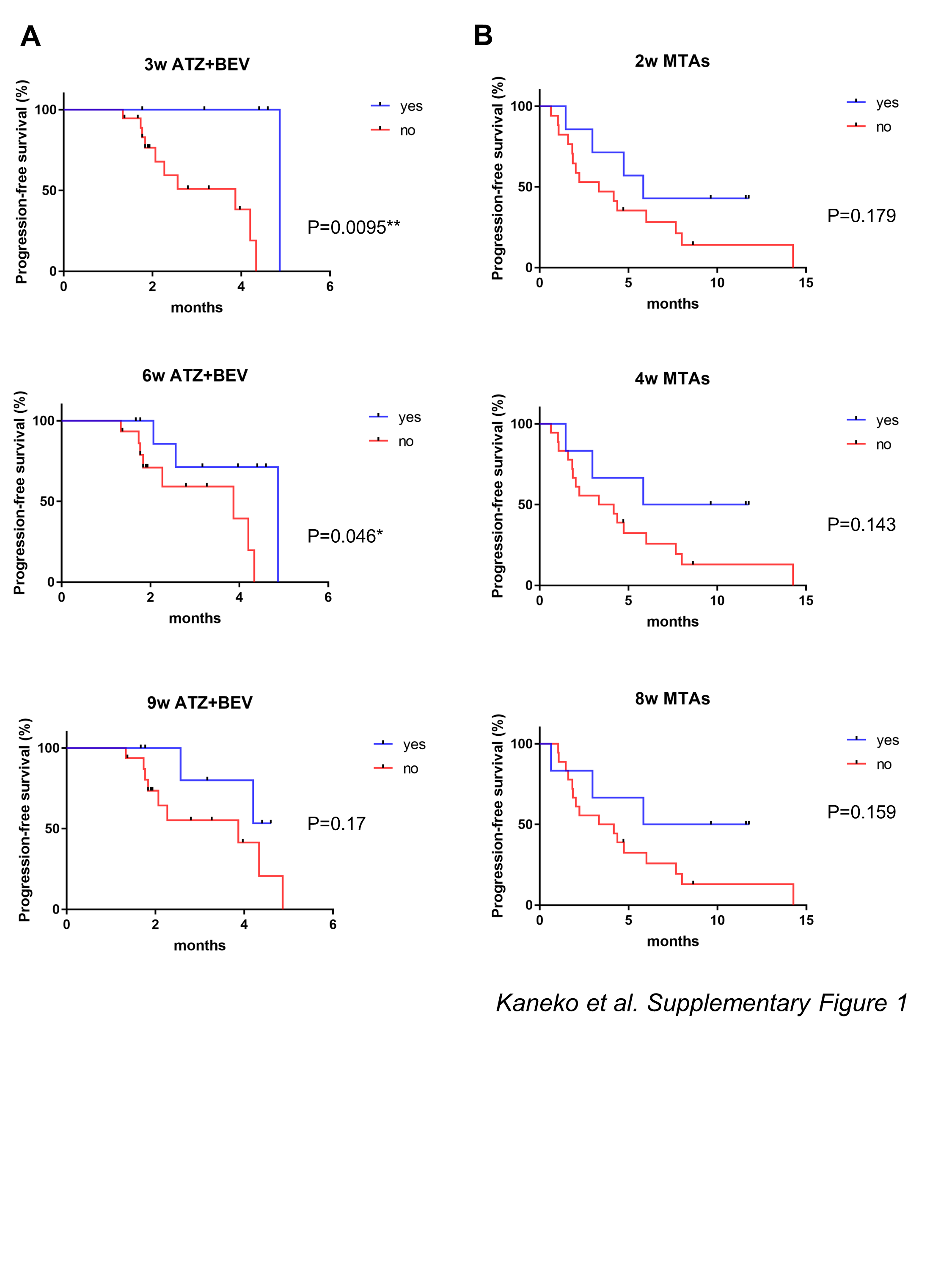

Supplement: S1 Fig — (A) PFS of patients treated by ATZ+BEV depending on Elecsys PIVKA-II response at 3, 6, and 9 weeks. (B) PFS of patients treated by MTAs depending on Elecsys PIVKA-II response at 2, 4, and 8 weeks. (TIF) [file pone.0265235.s001.tif]
